# Recurrence analysis on prostate cancer patients with Gleason score 7 using integrated histopathology whole-slide images and genomic data through deep neural networks

**DOI:** 10.1117/1.JMI.5.4.047501

**Published:** 2018-11-15

**Authors:** Jian Ren, Kubra Karagoz, Michael L. Gatza, Eric A. Singer, Evita Sadimin, David J. Foran, Xin Qi

**Affiliations:** a Rutgers, the State University of New Jersey, Department of Electrical and Computer Engineering, Piscataway, New Jersey, United States; b Rutgers Cancer Institute of New Jersey, Department of Radiation Oncology, New Brunswick, New Jersey, United States; c Rutgers Cancer Institute of New Jersey, Section of Urologic Oncology, New Brunswick, New Jersey, United States; d Rutgers Cancer Institute of New Jersey, Department of Pathology and Laboratory Medicine, New Brunswick, New Jersey, United States

**Keywords:** prostate cancer, Gleason score, deep neural networks, whole-slide images, genomic data

## Abstract

Prostate cancer is the most common nonskin-related cancer, affecting one in seven men in the United States. Gleason score, a sum of the primary and secondary Gleason patterns, is one of the best predictors of prostate cancer outcomes. Recently, significant progress has been made in molecular subtyping prostate cancer through the use of genomic sequencing. It has been established that prostate cancer patients presented with a Gleason score 7 show heterogeneity in both disease recurrence and survival. We built a unified system using publicly available whole-slide images and genomic data of histopathology specimens through deep neural networks to identify a set of computational biomarkers. Using a survival model, the experimental results on the public prostate dataset showed that the computational biomarkers extracted by our approach had hazard ratio as 5.73 and C-index as 0.74, which were higher than standard clinical prognostic factors and other engineered image texture features. Collectively, the results of this study highlight the important role of neural network analysis of prostate cancer and the potential of such approaches in other precision medicine applications.

## 1 Introduction

Prostate cancer remains the most common noncutaneous malignant tumor in the Western world accounting for approximately one in five of newly diagnosed tumors in men and resulting in an estimated 29,430 deaths in 2018.^1^ In the United States, approximately one in seven men will be diagnosed with this disease.^1^ Based on Gleason score, prostate specific antigen (PSA) value, tumor stage, age, and race, patients with prostate cancer are stratified into low-, intermediate-, and high-risk groups.^2^^,^^3^

A strong predictor of survival among men with prostate cancer is the Gleason score rendered by a pathologist based upon a microscopic evaluation of a representative histopathology specimen.^4^ These scores are based solely upon morphology and structural patterns of the constituent cells and glands. Patients with Gleason score 6 or lower often undergo active surveillance as there is reduced risk of tumor progression for those patients compared to patients with score 7 or higher.^5^^,^^6^ Tumors that are assigned Gleason score 7 can be delineated into a primary region exhibiting a histopathology pattern graded as 4 and a secondary region exhibiting a histopathology pattern graded as 3. Such samples are referred to as Gleason 4+3 tumors, whereas the inverse pattern exhibiting a primary pattern of 3 and a secondary pattern of 4 would constitute a Gleason 3+4 tumors. Patients with Gleason 4+3 tumors have an increased risk of recurrence and progression leading to an increased risk of prostate cancer-specific mortality when compared to those afflicted with Gleason 3+4 tumors.^7^^–^^9^ The literature clearly shows that predicting disease recurrence in a man with Gleason score 7 prostate cancer can have a significant impact on his disease management and survival.^8^^–^^10^

Phenotypically, tumor regions with Gleason pattern 3 are composed of single glands with distinct size and shape whereas ones with Gleason pattern 4 exhibit large irregular cribriform glands or fused, ill-defined glands with poorly formed glandular lumina.^11^^–^^13^ In spite of established guidelines, Gleason grading remains a relatively subjective process that results in an ∼30% grading discrepancy among the scores provided by pathologists.^11^^–^^15^ There have been many attempts to develop computer-aided Gleason grading methods and systems^11^^,^^16^^–^^21^ in order to introduce objective, reproducible criteria into the process of Gleason pattern quantification, and grading. One previous study has explored an integration of image features along with protein expression to predict recurrent prostate cancer.^22^ However, to date, there has been no study focused on utilizing patients’ pathology images and genomic pathway analyses in combination to predict recurrence-free survival (RFS) for men with prostate cancer.

Microarray-based gene expression signatures have been used in various studies to identify cancer subtypes, determine the RFS of disease, and characterize response to specific therapies.^23^ Multiple investigations have also shown that gene expression signatures can be used to analyze oncogenic pathways and these signatures have been used to identify differences between specific cancer types and tumor subtypes. Moreover, patterns of oncogenic pathway activity have been used to identify differences in underlying molecular mechanisms and have been shown to correlate with clinical outcomes of patients afflicted with specific cancers.^24^^–^^27^

In recent years, whole-slide image (WSI) has been more widely used in histopathology diagnosis. With a fast development of deep learning, histopathology image analysis approaches have demonstrated significant advances in cellular segmentation^28^^–^^31^ and tissue classifications^30^^–^^34^ using convolutional neural networks (CNN). Some research groups reported their studies using histopathology WSI for many applications.^35^^–^^37^ Due to a giga-pixel size of a WSI’s, it is often impractical to train the CNN using WSIs directly. Consequently, patch-based algorithms are widely applied in histopathology image analysis.^34^^,^^38^^–^^43^

In this study, we developed a computational biomarker quantification system by integrating histopathology WSIs and genomic data into one deep neural network. In order to use the distribution of Gleason patterns on a WSI, we applied patches as inputs to the network. The patches were forwarded through a CNN to get the images features. Then based on the patches’ spatial relationship, the image features were modeled using a recurrence neural network (RNN),^44^ namely long short-term memory (LSTM).^45^ The pathway scores calculated from the genomic data were forwarded to a multilayer perceptron (MLP) to get the genomic features. And the image and genomic features were integrated together to get the computational biomarkers. Moreover, we used RFS (months) since their initial treatment as the time-to-recurrence variable for a survival model. We chose a Cox proportional-hazard regression model^46^^,^^47^ since it is commonly used in medical research for investigating associations between the survival time of patients and predictor variables.

## 2 Methods

**Fig. 1:**
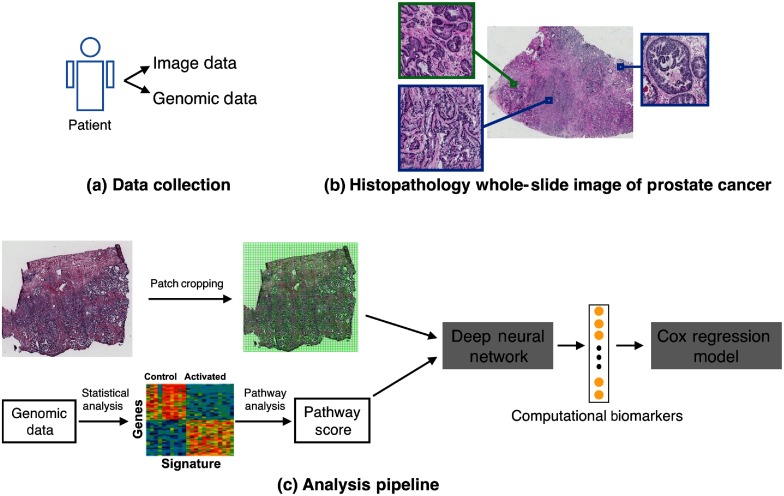
An overview of the pipeline of our study using histopathology WSIs and genomic data for prostate cancer recurrence prediction for patients with Gleason score 7. (a) WSI images and genomic data were collected from patients with prostate cancer; (b) a prostate WSI exhibits different Gleason patterns. For example, a region in a green square has the Gleason pattern 3 while regions in blue squares have the Gleason pattern 4; (c) the pathway scores were quantified using RNA sequences. Patches of region of interests were automatically selected from WSIs. The image patches and pathway scores were integrated into deep neural networks to extract computational biomarkers, which were fed into a Cox regression model in conjunction with clinical prognostic factors for disease recurrence analysis.

In this section, we introduced our approach on building a unified system using WSI and genomic data through deep neural networks to quantify computational biomarkers, which were fed into a survival model for patients’ recurrence analysis. Our methods consisted of four steps. First, the pathway activities of prostate cancer were quantified by pathway scores using RNA sequences. Second, the histopathology WSIs were preprocessed to obtain the region-of-interest as the image patches preparation. Third, the image patches and pathway scores were integrated into a unified system using the deep learning approach to extract computational biomarkers. Finally, we used the computational biomarkers in conjunction with clinical prognostic factors as the input of the survival model to calculate the disease recurrence ratios and probabilities. Figure 1 shows the overview of the pipeline of the whole study.

### 2.1 Experiment Dataset

In this study, we used publicly available prostate cancer data downloaded from the data portal of the Genomic Data Commons (GDC).^48^ GDC is the largest public available data portal that includes image data from The Cancer Genome Atlas (TCGA),^49^ genomic data, and clinical data. The TCGA barcode^48^ is the primary identifier of GDC data acquisition protocol. For this study, in total, there were 43 Gleason 3+3, 146 Gleason 3+4, 101 Gleason 4+3, and 49 Gleason 4+4, which contain 1229, 4753, 2997, and 1597 patches, respectively. For the recurrence study of patients with Gleason 7, we used all the data from Gleason 6, 7, and 8 to train the networks to extract the computational biomarkers. In this way, the training data contained more images of Gleason patterns 3 and 4 compared to a training data if only use patients’ data with Gleason 7 (3+4 or 4+3). For the recurrence study of patients with Gleason 7, the computational biomarkers of patients with Gleason 7 were fed into a survival model while the patients with other Gleason score were withheld.

The patients were randomly divided into the training set, validation set, and testing test with the ratio of 70%, 10%, and 20%; these groups were utilized for the recurrence analyses. In addition to the Gleason score, we compared the computational biomarkers quantified from the unified image and genomic data system with other clinical factors including patients’ PSA, age, and tumor stage, which are publicly available from GDC data portal.

The WSI patches preparation was a two-step cropping-selection process. First, the image patches within each WSI were automatically cropped under 40× objective magnification with a patch size 4096×4096. The patches were cropped with a stride as 4096 to avoid overlapping. We resized all the patches to the size of 256×256 using Lanczos filtering.^50^ Second, any specimens with insufficient tissue patches were automatically eliminated from the experiments due to the heterogeneous quality of the prostate WSIs. The patches with the tissue accounting for at least 20% of the whole area were selected.

### 2.2 Pathway Score Quantification from RNA Sequencing Data

To quantify pathway scores, we used the gene expression data, which were RNA (Illumina HiSeq) sequencing data from patients with Gleason score 7. The data are publicly available through GDC data portal. We preprocessed the RNA data by log transformed and median centered. A panel of previously published 265 experimentally derived gene expression signatures was applied to the entire cohort to identify patterns of oncogenic signaling in each tumor. To apply a given signature, the expression data were filtered to contain only those genes included in the given signature and the mean expression value of these genes was calculated to provide a score for each sample.^25^^,^^26^

### 2.3 Computational Biomarker Extraction

**Fig. 2:**
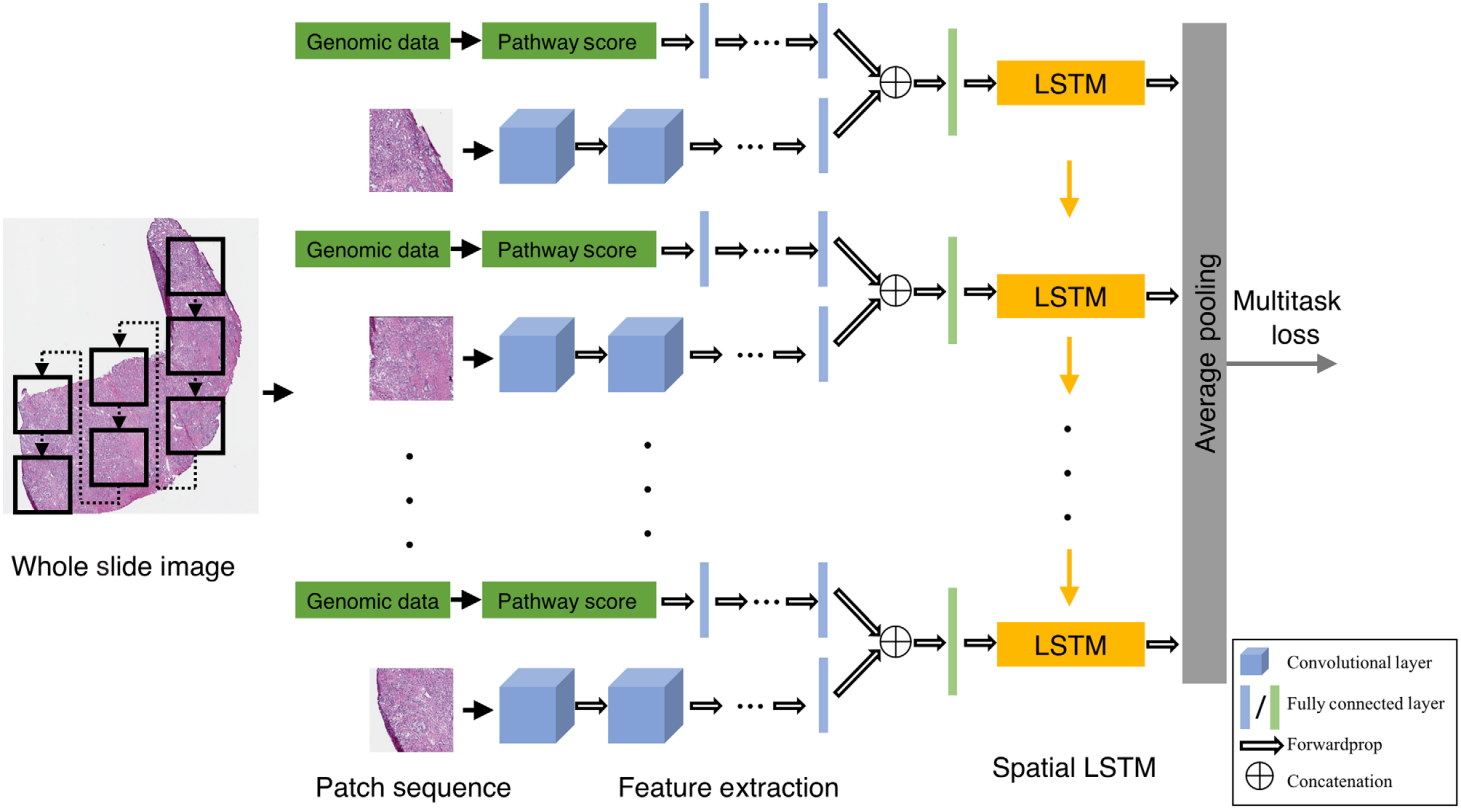
Network architecture for extracting computational biomarkers from the WSI and genomic data. We used seven LSTM cells in the network. The calculated pathway scores from the genomic data were forwarded into an MLP that contains three FC layers. The last layer of the MLP was connected with the features extracted from the image patches to serve as the input for the LSTM after an FC layer. On top of the LSTM, we utilized an average pooling layer.

In order to obtain computational biomarkers from the WSIs and genomic data, we built a unified feature quantification system using CNN to model WSI histopathology image patches and genomic data together. Furthermore, we leveraged the RNN to model the spatial relationship of the cropped patches within the WSI. The network architecture is shown in Fig. 2.

#### 2.3.1 Modeling histopathology image patches and genomic data

In order to combine the image information along with the genomic data, we used the patches and pathway scores as the input to the network. We forwarded the pathway scores into an MLP that includes three fully connected (FC) layers, with 1024, 512, and 256 hidden units, respectively. The genomic features were the output of the last FC layer. Meanwhile, we incorporated the AlexNet^51^ to extract the features from image patches. We concatenated the genomic features obtained from the pathway scores with the image features from the second to the last layer of the AlexNet. The concatenated features served as the input to an FC layer before LSTM.

Due to the giga-pixel WSI’s, we considered an integrity of the whole tissue regions on a single WSI instead of using the individual patches to quantify image features as shown in previous studies.^39^^,^^41^ The spatial relationship of the adjacent patches was modeled as an image sequence. We adopted a type of RNN,^44^ LSTM,^45^ to model the features extracted from the image patches and genomic data given LSTM has shown its successes among various applications including speech recognition,^52^^,^^53^ language translation models,^54^ image captioning,^55^ and video classification.^56^ Compared with the traditional RNN that has vanishing and exploding gradients problems, LSTM is more effectively in sequence modeling by incorporating memory cells with several gates to obtain long-range dependencies.

**Fig. 3:**
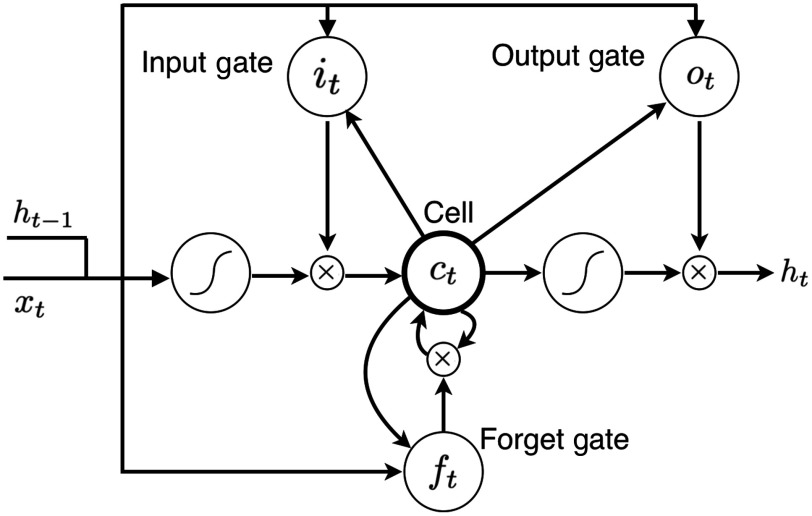
The visualization of an LSTM cell.

More formally, for the input feature sequence ($x_{1},x_{2},\ldots ,x_{T}$) that $x_{i}$ represents the activations from the CNN of the i’th patch, we used LSTM to compute the output sequence ($y_{1},y_{2},\ldots ,y_{T}$), where the layer of LSTM was computed recursively from t=1 to t=T following the equations:

$$i_{t}=\sigma (W_{xi}x_{t}+W_{hi}h_{t-1}+W_{ci}c_{t-1}+b_{i}),$$ (1)

$$f_{t}=\sigma (W_{xf}x_{t}+W_{hf}h_{t-1}+W_{cf}c_{t-1}+b_{f}),$$ (2)

$$c_{t}=f_{t}c_{t-1}+i_{t}\;\tanh(W_{xc}x_{t}+W_{hc}h_{t-1}+b_{c}),$$ (3)

$$o_{t}=\sigma (W_{\mathrm{xo}}x_{t}+W_{\mathrm{ho}}h_{t-1}+W_{\mathrm{co}}c_{t}+b_{o}),$$ (4)

$$h_{t}=o_{t}\tanh(c_{t}),$$ (5)

where $h_{t}$ is the hidden vector, $i_{t}$, $c_{t}$, $f_{t}$, and $o_{t}$ represent the activation vectors of the input gate, memory cell, forget gate, and output gate, respectively. W terms denote the weight matrices connecting different units, b terms denote the bias vectors, and σ is the logistic sigmoid function. The memory cell $c_{i}$ has the inputs of the weighted sum of the current inputs and the previous memory cell unit $c_{t-1}$, which could learn when to forget the old information and when to consider the new information. The output gate $o_{t}$ controls the propagation of information to the following step. The visualization of the LSTM cell is shown in Fig. 3.

Since it is a sequential task to train LSTM, patches from a WSI were formed by a specific routine. As shown in Fig. 2, we used center coordinates of patches to remark the location of each patch. The sequence of patches within a single WSI was arranged from right up patch down to lower left one, which was illustrated by the dotted lines with black arrows on an example of a WSI on Fig. 2. In this way, it allowed us to consider both unique characteristics of each patch and fine-grained variations among patches within a single WSI. For each tumor WSI, the patches and the pathway scores were fed into the network to get features and then incorporated into the LSTM recursively. In addition, the average pooling layer was applied on top of the network to get the computational biomarkers for the WSI and the genomic data. The number of hidden units for each LSTM was 1024. During the training process, we applied the multitasks loss and assigned the primary pattern and the Gleason score for the WSIs and genomic data.

#### 2.3.2 Multitask loss function

For the TCGA prostate WSIs, the primary Gleason pattern, the secondary Gleason pattern, and the sum of both patterns (i.e., Gleason score) were publicly available from GDC data portal. To model the variations among Gleason patterns, we utilized the multitask loss to enable the network to learn as much information about the Gleason pattern distributions from the patches of a WSI as possible. Therefore, we gave the primary pattern and the sum score as labels for each patch along with the pathway score and use the following multitask loss function:

Lmultitask=−∑i=0Ntip·log t^ip−∑i=0Ntis·log t^is, (6)

where for the i’th input sample within the batch of N samples, $t_{i}^{p}$ and $t_{i}^{s}$ are the one-hot encoding of the Gleason grading for the primary pattern and the sum score, respectively, t^ip and t^is are the predicted grading of the model.

### 2.4 Survival Model

In conjunction with clinical prognostic factors including the primary and secondary Gleason patterns, PSA, age, and tumor stage, computational biomarkers were fed into a Cox regression model^46^^,^^47^ for studying patient’s RFS. In our study, we used RFS (months) as the time variable for a survival model. For high dimensional data, only those with Wald test^57^^,^^58^ p-value <0.05 were selected and used in conjunction with clinical prognostic factors as input variables for the Cox regression model.

One of the most popular regression techniques for survival analysis is Cox proportional hazards regression, which is used to relate several risk factors or exposures, considered simultaneously, to assess differences in overall survival. In a Cox proportional hazards regression model, the measure of effect is the hazard ratio, which is the risk of failure (i.e., here is the risk or probability of the recurrence of the disease), given that the participant has survived up to a specific time. Given the patients ($t_{i}$, $l_{i}$, $X_{i}$), where i=1,2,…,N, we have the $t_{i}$ as the patient’s recurrence time for individual i; $l_{i}$ as the label of the censored data that equals 1 if the recurrence occurred at that time and 0 if the patient has been censored; $X_{i}$ as the vector of covariates of the selected image features and clinical factors. The hazard function is the nonparametric part of the Cox proportional hazards regression function corresponding to

$$H(X_{i},l_{i},t_{i})=H_{0}(t)\exp\overset{p}{\underset{j=1}{\sum }}x_{ij}\beta_{i}.$$ (7)

Here, $x_{ij}$ is the computational biomarkers j for patient i, where j=1,2,…p and $\beta_{i}$ is the Cox regression parameter for each patient. Here, $H_{0}$ is the baseline hazard function. The hazard ratio is derived from $\mathrm{HR}(X_{i})=\frac{H(X_{i},l_{i},t)}{H_{0}}$, representing the relative risk of instant failure for patients having the predictive value $X_{i}$ compared to the ones having the baseline values. Here, $d_{i}$ is weighting parameters for each patient:

$$\mathrm{HR}(X_{i})=\overset{N}{\underset{i}{\sum }}d_{i}\{X_{i}\beta_{i}-\log[\overset{p}{\underset{j}{\sum }}I(t_{j}-t_{i})\exp(X_{i}\beta_{i})]\}.$$ (8)

In the study, we assessed the computational biomarkers in conjunction with other clinical prognostic factors by their recurrence hazard ratios and concordance indices (C-index).^59^^,^^60^ The hazard ratio and C-index both are global indices for validating the predictive ability of prognostic features of a given survival model. Under a given survival model, higher values mean that prognostic features predict higher risks and probabilities of survival for higher observed survival times. In our study, we examined RFS; the higher the hazard ratio and C-index, the greater the likelihood of disease recurrence.

## 3 Experiments and Results

In this section, we validated our approach on a publicly available prostate cancer dataset from the GDC data portal. The experimental results showed that the computational biomarkers discovered by the proposed method were effective for recurrence correlation for patients with Gleason score 7.

### 3.1 Implementation Details

The training process of our network included two steps. We first trained the CNN using minibatch Stochastic gradient descent with batch size as 32, momentum as 0.9, and weight-decay as $5\times 10^{-5}$. The initial learning rate was $10^{-3}$ and annealed by 0.1 after every 10,000 iterations. We trained the CNN for total of 50,000 iterations until the loss converge. Then, we utilized the genomic data to train the MLP to extract the genomic features and used image and genomic features to train LSTM. We kept the same momentum, weight-decay, and learning rate except, we annealed the learning rate by 0.1 after every 2000 iterations and trained the network for a total of 5000 iterations. The implementation was based on Caffe toolbox.^61^

### 3.2 Pathway Analysis

Multiple studies have shown that gene expression signatures reflect the activation status of oncogenic pathways irrespective of specific mutations driving signaling.^24^^–^^26^ Thus, we examined genomic-based patterns of oncogenic pathway activity in prostate cancer patients with Gleason score 7 using a panel of previously published 265 gene expression signatures.

**Fig. 4:**
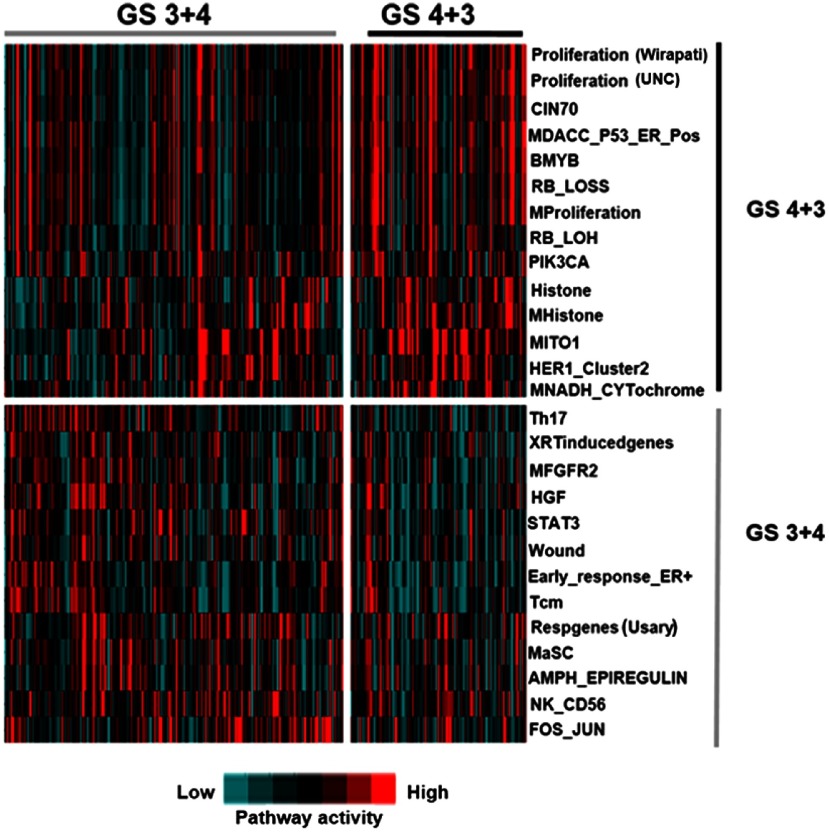
Differential patterns of pathway activity in Gleason score 3+4 and 4+3 prostate tumors. Comparative analysis of Gleason score 4+3 (n=101) and Gleason score 3+4 (n=146) tumors identified 27 significantly altered signaling pathways (t-test, p<0.01) as defined by mRNA-based gene expression signature scores. Tumors with a Gleason score 4+3 showed higher proliferation, BMYB, RB-LOH, and histone modification signature scores while tumors with a Gleason score 3+4 showed higher levels of immune system related pathway signatures including Th17 cells, Tcm, and STAT3.

In order to qualitatively assess unique patterns of pathway activity that define the 4+3 and 3+4 subset of Gleason score seven tumors, pathway signatures in each group, using all tumors across the entire cohort (i.e., training, test, and validation tumors) were assessed by a Student’s two tailed t-test. Significant pathway scores were clustered using Cluster 3.0^62^ and visualized by Java TreeView.^63^ Quantitative assessment of patterns of pathway activity of Gleason score 4+3 and 3+4 subgroups is shown in Fig. 4, which displayed a heatmap identifying 27 differentially expressed signatures (p<0.01). Of these, we determined that 14 signatures including three unique proliferation signatures (Wirapati,^64^ UNC,^65^ and murine proliferation^65^) as well as several proliferation-associated signatures predicative of BMYB,^66^ RB-LOSS,^67^ PIK3CA,^68^ and HERI^69^ signaling were significantly higher in patients with Gleason score 4+3. We further determined that 13 signatures were upregulated in Gleason 3+4 patients including immune systems signatures associated with Th17 cells,^70^ Tcm,^70^ NK-CD56,^70^ HGF,^71^ and STAT3^26^ signaling. Consistent with our findings, many studies report^72^^–^^74^ that Gleason 3+4 tumors have a better prognosis than Gleason 4+3 tumors, which would correlate with relatively higher levels of proliferation as well as lower levels of immune-related signaling evident in Gleason 4+3 tumors compared to Gleason 3+4 samples.

### 3.3 Integrated Recurrence Analysis in Conjunction with Clinical Factors

#### 3.3.1 Image data on recurrence analysis

For the integrated recurrence analysis using a survival model, we first conducted the experiments where only the WSIs of tissue slides were used. Thus, the networks were trained without integrating the genomic features. This setting of experiment is denoted as CNN-LSTM. We also considered the setting that only CNN was applied on the image patches without considering their spatial relation on a WSI and the image features were extracted from the second to the last layer of AlexNet. The setting is denoted as CNN-Only. To compare the effectiveness of the feature extraction from the images, we applied three texture feature methods including SURF,^75^ HOG,^76^ and LBP^77^ on the WSIs to obtain image features. The image features were concatenated with clinical prognostic factors as multivariate inputs of the Cox regression model. During each iteration, each image feature in conjunction with clinical factors was fed into the Cox regression model to calculate the corresponding hazard ratios and C-indices. The survival model implementation was based on an R survival package.^78^

The maximum hazard ratios of recurrence of image features in conjunction with clinical factors are shown in Table 1. Within our study, we used the disease RFS times as the time variable in the Cox regression model, the higher values of hazard ratio and C-index of the features indicated that the image features had the higher correlations with the disease recurrence and progression. From the result of using texture features, there were no significance differences among LBP, HOG, and SURF for recurrence ratios. CNN-LSTM analysis determined that image features identified by computational image analysis outperformed other texture features and CNN-Only with higher hazard ratio and C-index. When conjunction with CNN-LSMT, the primary pattern still showed greater hazard ratio and C-index relative to those identified using other methods.

**Table 1:** Recurrence hazard ratios and corresponding C-indices of clinical prognostic factors and different image features from various image quantification methods. The results are obtained by using image features quantified from the WSIs. LBP, HOG, and SURF are the texture methods. CNN-LSTM is using the image features obtained from CNN with LSTM while CNN-Only is using the image features obtained from CNN without considering patches’ spatial relation on a WSI.

Methods	Primary pattern	Secondary pattern	PSA	Age	Tumor stage	Image features	C-index
LBP	1.05	0.94	0.85	1.00	1.03	1.05	0.68
HOG	1.04	0.94	0.85	1.00	1.03	1.05	0.64
SURF	1.07	0.97	0.86	1.00	1.03	1.05	0.61
CNN-Only	1.11	1.12	0.80	1.00	1.17	2.44	0.70
CNN-LSTM	1.70	1.06	0.80	0.99	1.26	5.06	0.71

#### 3.3.2 Image and genomic data on recurrence analysis

Before integrating image features and pathway scores, we first analyzed the correlation between them. Because the number of image features and the number of pathway scores were different, to calculate their correlation coefficients, we randomly chose the same number of image features paired with the same number of pathway scores and repeated the process N times until all image features had been paired. Here, the image features included features quantified from texture methods (LBP, HOG, and SURF) and CNN-LSTM. Using a t-test on correlation coefficients, the mean and standard deviation of p-values is shown in Table 2. Because p-value >0.05, there was no significant correlations between image features and pathway scores. This showed that the two types of data provided complementary information for prostate cancer diagnosis and prognosis. It was reasonable to integrate image and genomic data together for predicting patients’ recurrence.

**Table 2:** Correlation analysis of image features and pathways scores using a test-test on their correlation coefficients.

Image features	Mean of p-value	Standard deviation of p-value
LBP	0.50	0.29
HOG	0.49	0.30
SURF	0.43	0.30
CNN-LSTM	0.48	0.29

Then, we showed the experimental results by combining image features obtained from WSIs and the genomic features obtained from the pathway scores. We utilized all 265 gene expression signatures integrated with image data to identify the computational biomarkers as shown in Fig. 2. The setting was denoted as CNN-LSTM + PS. We also considered the setting where LSTM was deactivated when obtained the biomarkers from image and genomic data. We denoted the approach as CNN-Only + PS. The methods using texture features obtained from WSIs together with pathway scores for the recurrence analysis were denoted as LBP-PS, HOG-PS, and SURF-PS. We also considered only using pathway scores with clinical factors together as the input of the Cox regression model and denote it as PS. The maximum hazard ratios of the computational biomarkers from WSIs and pathway scores in conjunction with clinical factors are shown in Table 3.

**Table 3:** Recurrence hazard ratios and corresponding C-indices of clinical prognostic factors and computational biomarkers under a Cox regression model using different image feature quantification methods along with the genomic data. Given the genomic data, we show the results using image features with pathway scores (PS). Here, LBP + PS, HOG + PS, SURF + PS, CNN-Only + PS, and CNN-LSTM + PS are image features quantified from LBP, HOG, SURF, CNN-Only, and CNN-LSTM methods with PS.

Methods	Primary pattern	Secondary pattern	PSA	Age	Tumor stage	Biomarkers	C-index for biomarkers
PS	0.95	0.98	0.86	1.00	1.04	1.02	0.65
LBP + PS	1.04	1.00	0.87	1.00	1.02	1.08	0.69
HOG + PS	1.04	1.00	0.87	1.00	1.02	1.08	0.65
SURF + PS	1.07	1.00	0.86	1.00	1.03	1.07	0.62
CNN-Only + PS	1.13	1.11	0.80	1.00	1.17	2.58	0.71
CNN-LSTM + PS	2.56	0.63	0.66	1.01	1.05	5.73	0.74
C-index for clinical factors	0.61	0.59	0.66	0.55	0.53	—	—

Compared with other clinical factors, using pathway scores alone achieved equivalent hazard ratio. For the texture methods, the recurrence hazard ratios were equivalent to the ones without pathway scores. Using CNN-LSTM + PS, the Gleason primary pattern and computational biomarkers showed the increased recurrence ratios compared to the ones without pathway scores. In addition, the Gleason primary pattern and computational biomarkers showed the highest recurrence ratios compared to other clinical factors. The result showed that CNN-LSTM-PS outperformed other methods in prostate cancer recurrence analysis due to its highest recurrence hazard ratio.

Furthermore, we show the C-index of the clinical factors and computational features under the Cox regression model for prostate cancer recurrence probability prediction in the last column of Table 1 and the last row and column of Table 3. As a global index for validating the predictive ability of a survival model, in our study, the C-index was equivalent to a rank correlation of the risk of a recurrence of disease. High values mean that the model predicts higher probabilities of recurrence for higher observed recurrence times. From the clinical results, PSA showed higher C-index values, which were correlated to a higher recurrence prediction probability compared to other clinical factors. Interestingly, texture features on WSIs or pathway scores individually showed an equivalent recurrence probability.

## 4 Discussion

From the experimental results, our proposed method achieved the highest recurrence hazard ratio and the strongest *C*-index related to prostate cancer recurrence probability compared to other clinical prognostic factors and methods. It demonstrated that the approach was beneficial for recurrence analysis on the patients with Gleason score 7. The unified WSIs and genomic data analysis through the proposed networks could be applied to other prostate cancer risk group such as Gleason 6^79^^–^^81^ or other cancer recurrence analysis, such as breast cancer.^82^

Pathway analysis, albeit with the caveat of a small sample size, identified 27 differentially expressed pathway activities in tumors with Gleason score 3+4 and 4+3. Thus, these signatures could be utilized to differentiate patients with Gleason score 7 as two subgroups, which corresponds with a favorable or unfavorable prognosis.^83^ The recurrence analysis (Table 3) using pathway scores alone did not show an advantage over other clinical prognostic factors. The integration of pathway score with WSIs achieved the best recurrence prediction on patients with Gleason score 7. The comparison indicated that using the pathway scores directly had a limited contribution in recurrence prediction on patients with Gleason score 7. However, the embedded genomic features obtained through MLP were more effective for prostate cancer recurrence analysis.

There are other clinical factors for prostate cancer prognosis besides those used in the study, such as patients’ race. Because in the study, <2% men were Asian or African, 30% were Caucasian, and the rest were unknown, we excluded patients’ race factor in the recurrence analysis. Other clinical factors, such as American joint committee on cancer metastasis stage, neoplasm disease stage codes, and so on, were not available for all the patients in the GDC prostate cancer datasets.

The prostate cancer datasets were acquired from various institutions and each institution may have different scanners or WSI scanning protocols. Thus, there was color heterogeneity among the prostate cancer WSIs. In the study, we did not adopt color normalization^84^^,^^85^ on the randomly selected testing set because it was not feasible to determine the reference image from the training set for color normalization. When we apply the approach to a new dataset, we could fine-tune the network based on the training data from that dataset. Given the limited size of the public prostate dataset, the results achieved from our experiments were preliminary. In order to further validate the generalizability of our approach on a wider population of prostate cancer patients, we will collect more prostate images from local institutions to perform extensive experiments.

## 5 Conclusion

In this study, we performed recurrence analyses for prostate cancer patients with Gleason score 7 integrating histopathology WSIs and genomic data. The image features and genomic features were obtained using CNN and MLP, respectively. The combination of the features was modeled using LSTM to get the computational biomarkers. Experimental results utilizing on publicly available prostate cancer dataset showed that the computational biomarkers extracted by our approach were more closely correlated with patients recurrence risk compared to standard clinical prognostic factors and engineered image texture features. The results of our study suggest that these approaches could be utilized to predict recurrence and progression for patients with prostate cancer.
